# Single-centre survival analysis over 10 years after MR-guided radiofrequency ablation of liver metastases from different tumour entities

**DOI:** 10.1186/s13244-022-01178-8

**Published:** 2022-03-21

**Authors:** Susann-Cathrin Olthof, Daniel Wessling, Moritz T. Winkelmann, Hansjörg Rempp, Konstantin Nikolaou, Rüdiger Hoffmann, Stephan Clasen

**Affiliations:** 1 Department of Diagnostic and Interventional Radiology, University Hospital of Tuebingen, Hoppe-Seyler-Straße 3, 72076 Tuebingen, Germany; 2 Radiologie Waiblingen, Alter Postplatz 2, 71332 Waiblingen, Germany; 3 Department of Radiology, Kreiskliniken Reutlingen, Steinenbergstraße 31, 72764 Reutlingen, Germany

**Keywords:** MR-based radiofrequency ablation, Minimal invasive tumour ablation, Long time overall survival

## Abstract

**Background:**

Radiofrequency ablation (RFA) is a minimal-invasive, local therapy in patients with circumscribed metastatic disease. Although widely used, long time survival analysis of treated liver metastases is still pending while also analysing the patients’ experience of MR-based radiofrequency.

**Methods:**

Monocentric, retrospective analysis of long-time overall and progression free survival (OS; PFS) of 109 patients, treated with MRI-guided hepatic RFA between 1997 and 2010, focusing on colorectal cancer patients (CRC). Complimentary therapies were evaluated and Kaplan Meier-curves were calculated. Patients’ experience of RFA was retrospectively assessed in 28 patients.

**Results:**

1-, 3-, 5-, 10-year OS rates of 109 patients with different tumour entities were 83.4%, 53.4%, 31.0% and 22.9%, median 39.2 months, with decreasing survival rates for larger metastases size. For 72 CRC patients 1-, 3-, 5-, 10-year OS rates of 90.2%, 57.1%, 36.1% and 26.5% were documented (median 39.5 months). Thereof, beneficial outcome was detected for patients with prior surgery of the CRC including chemotherapy (median 53.0 months), and for liver metastases up to 19 mm (28.5% after 145 months). Hepatic PFS was significantly higher in patients with liver lesions up to 29 mm compared to larger ones (*p* = 0.035). 15/28 patients remembered RFA less incriminatory than other applied therapies.

**Conclusions:**

This is the first single-centre, long-time OS and PFS analysis of MRI-guided hepatic RFA of liver metastases from different tumour entities, serving as basis for further comparison studies. Patients’ experience of MR based RFA should be analysed simultaneously to the performed RFA in the future.

## Key points

- MR-guided RFA for liver metastases offers a 10-year survival rate of 22.9%
- Thereof 10-year survival data of 72 colorectal cancer patients was 26.5%
- 15/28 patients stated RFA to be less incriminatory than other applied therapies

## Background

The liver is the organ which is most commonly affected from distant metastases of any malignant tumour, due to the double vascular supply through the hepatic artery and portal vein [1]. Among potential curative surgical options, interventional therapies like microwave ablation (MWA) and radiofrequency ablation (RFA) are also providing a locally effective treatment by induction of necrosis in hepatic metastasis. The main advantages of the minimally invasive RFA encompass insufficient functional liver resources and comorbidities disenabling any surgical therapy [2]. CT-, MR-, sonography-and even PET-based RFA procedures of the liver are technical possible [3, 4]. MR-guided RFA is routinely applied in our department as it provides precise delineation of the tumour area based on a high tissue contrast [5]. Furthermore, accurate and safe needle position including monitoring of therapeutic efficacy using T1w sequences is possible without any repetitive i.v. contrast application like in CT guided interventions [6, 7].

Comparable 5 year survival rates of 33% are reported for surgical- and RFA treatment in 309 colorectal patients with up to three liver metastasis of a max. diameter of 3.5 cm, if the metastasis is ablated completely [8]. Furthermore, local recurrence rate between both therapeutic options is similar for small metastatic lesions ≤ 3 cm and tumour margins > 5 mm in CRC patients [9]. However, for larger metastatic CRC lesions, RFA local recurrence rates are reported between 2 and 60% [10].

In contrast to the aforementioned literature either focusing on laparatomic, laparascopic, ultrasound- and CT-guided RFA or on the technical innovation of MR-guided RFA, this study aims at the clinical long-term outcome after 10-years of transcutaneous MR-based RFA for hepatic metastases from different tumours, with a focus of colorectal metastases, including the analysis of patients’ satisfaction of this minimal invasive procedure.

## Methods

### Study design

In this retrospective, monocentric study, long time overall survival (OS) and progression free survival (PFS) of patients with hepatic metastases from different primary tumours, treated with MRI-guided hepatic RFA between 02/1997 and 5/2010, was analysed. A subgroup analysis was performed, according to patients’ primary diagnoses with emphasis on colorectal cancer (CRC). Additionally, the individual patients’ experience of the MR guided RFA was retrospectively assessed in 2020 in 28 patients.

### Data acquisition

Follow-up data of patients, basic demographic data and details about other applied therapies were extracted either from the in-house-recording system or through a telephone survey of the patients or their relatives. Additionally, a questionnaire including subjective strain of the applied RFA in comparison to other performed therapies was retrospectively applied for each patient within the scope of a “patient reported outcome” (PRO). The study was reviewed and approved by the local ethics committee.

### Patient cohort

168 patients, thereof 120 male and 48 female patients were treated with RFA for hepatic tumour lesions. Mean age at the time point of first RFA was 63.89 years (SD 10.76).

**Fig. 1:**
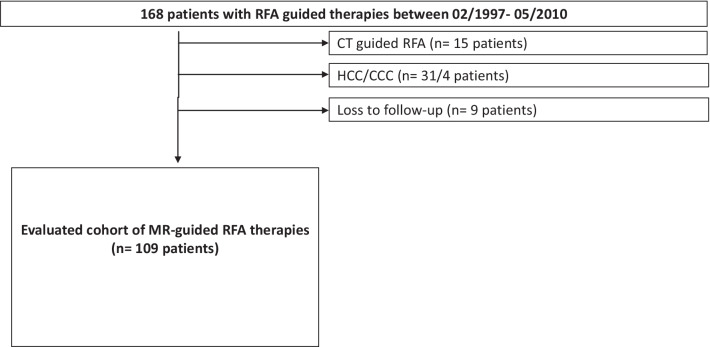
Overview of the whole study cohort with 109 included patients, treated with MRI based RFA for liver metastases

Inclusion criteria for RFA performance were hepatic metastasis up to 5 cm, no central liver lesions near any biliary structures or large portal veins and no MR contraindications. Exclusion criteria for study analysis were CT-guided RFA (*n* = 15), RFA in patients hepatocellular or cholangiocellular cancer (*n* = 31 and 4) or loss to follow-up (*n* = 9) resulting in 109 included patients (Fig. 1).

### MR-guided RFA procedure

RFA-therapy was planned on pre-interventional 1.5 T MRI. Percutaneous RFA procedures were performed between 1997-06/2006 with a 0.2 T MR scanner (Magnetom open, Siemens Healthineers, Erlangen, Germany, [11]) and from 07/2006–2010 with a 1.5 T MR scanner (Siemens Magnetom Espree, Siemens Healthineers) using both body array and loop array coils. RFA procedures were performed by the interventional group of our faculty with four core members either performing or surveilling the procedure. After patient positioning including placement of grounding pads on their back, pulse rate and oxygen saturation monitoring systems were installed, followed by a grid marking the interventional access path (TargoGrid™, Invivo Germany GmbH, Schwerin, Germany). According to interventions performed at respectively 0.2 T and 1.5 T different MR sequences were applied (Table 1).

**Table 1:** Overview of the different applied MR sequences for RFA performed at 0.2 T and 1.5 T

	0.2 T	1.5 T
MR planning examination: verification of lesion size and localisation	T1w SE sequence	HASTE
	T2w fast SE sequence	T1w in- and opp phase
		T1w Flash 2D
		T2w TSE
		DWI (EPI) (T1w VIBE)
Patient preparation	i.v. analgesia and sedation	
	s.c. local anaesthetics (Xylocaine 0.9%, AstraZeneca, Wedel, Germany)	
RF applicator placement: fluoroscopic sequences	fast T1w or T2*w GE sequences	Either T1w or T2w BEAT_IRTT (SSFP)
Interventional imaging	T2w fast SE sequences	T1w Flash 2D
		T2w TSE, T2w FISP
		DWI
		Breath-hold gated EPI sequence
Postinterventional imaging	T1w SE	T1w
	T2w fast SE	T2w
		T1w post contrast VIBE (0.1 mmol gadobutrol per kilogramm; Gadovist, Bayer Vital GmbH, Leverkusen, Germany)

Between 1997 and 06/2006 monopolar- (Valleylab, Covidien, USA), and between 07/2006 and 2010 bi-and multipolar RFA applicators were applied (Olypmus Celon, Teltow, Germany); for further details of the inhouse standardised RFA procedure see also [12].

Imaging follow-up was primarily based on MRI for the liver and CT for whole body staging, starting 1 month after RFA, subsequently followed every 3 months for 1 year and afterwards every 6 months. MR liver examinations for the assessment of the ablation zone including the presence of tumour-suspect tissue, were evaluated by experienced radiologists. Analysed issues included the size of the ablation zone, the location of the ablation zone in relation to the original tumour location as well as the initial signal behaviour of the tumour with signal changes adjacent to the ablation zone in T1w, T2w, diffusion-weighted sequences and post contrast enhanced T1w sequences [13, 14].

### Statistics

Continuous variables and frequencies are given as means with standard deviation (SD). Progression free survival included either occurrence of new liver metastases and local recurrence of the treated liver lesions (“hepatic PFS”), or new metastasis occurrence in the whole body (“extrahepatic PFS”). PFS was calculated from the date of the first imaging method stating metastases respectively in the liver and the whole body.

OS was defined as time between RFA-procedure and death, documented in inhouse medical records or query of the general physician or family members. Death was defined as a censoring event.

Both PFS and overall survival (OS) data were calculated applying Kaplan Meier curves in SPSS statistics (Version 27, IBM Corporation, Armonk, North Castle, NY) with median survival months including the standard deviation. For comparisons of OS and PFS according to subgroups, log-rank test was applied with a *p* value ≤ 0.05 considered as statistically significant.

## Results

### Patient characteristics

109 patients, thereof 79 male and 30 female patients were treated with MR-guided RFA between 02/1997 and 05/2010 suffering from hepatic metastases of different tumours (Table 2). Mean age was 63.5 years (SD 11.6). Mean time between primary tumour diagnosis and RFA was 2.6 years (SD ± 3.4). 88/109 patients (80.7%) died in the analysed follow-up period.

**Table 2:** Cohort characteristics of patients treated with RFA

Patients (total *n* = 109)		
Age	Years	± SD
Mean	63.5	± 11.6
Sex	No. of patients	%
Male	79	72.5
Female	30	27.5
Primary diagnoses (total *n* = 109)		
Colorectal carcinoma	72	66.1%
Melanoma	14	12.8%
Breast carcinoma	8	7.3%
Neuroendocrine tumours (NET)	5	4.6%
Bronchial carcinoma	1	0.9%
Urothelium carcinoma	1	0.9%
Renal carcinoma	2	1.8%
Gastric carcinoma	2	1.8%
Pancreatic carcinoma	1	0.9%
Gallbladder carcinoma	2	1.8%
Hypopharyngeal carcinoma	1	0.9%
Diameter of 244 treated liver metastases		
Mean	20.1 mm	± SD 10.0
< 3 cm	202	82.8%
≥ 3 cm	42	17.2%
Localisation of liver metastases		
Segment II	19	7.8%
Segment III	15	6.1%
Segment IV	54	22.1%
Segment V	28	11.5%
Segment VI	55	22.5%
Segment VII	33	13.5%
Segment VIII	40	16.4%
Number of RFA treated lesions per patient		
1 lesion	54 patients	45.5%
2 lesions	26 patients	23.9%
3 lesions	8 patients	7.3%
4 lesions	9 patients	8.3%
5 lesions	5 patients	4.6%
6 lesions	2 patients	1.8%
7 lesions	2 patients	1.8%
9 lesions	3 patients	2.8%

Total number of RFA-treated liver lesion was 244, thereof *n* = 1 in 54 patients and *n* = 2 in 26 patients; for further details see Table 2. Six patients were repetitively treated due to local disease recurrence on the ablation zone and 16 patients due disease recurrence in another liver segment.

The mean diameter of the largest liver metastases was 20.1 mm (SD 10.0 mm). Liver metastases ≥ 3 cm and ≤ 5 cm were treated in *n* = 42 patients (17.2%). Treated liver metastases were located mainly in segment IV and VI of the liver (22.1% and 22.5%). Colorectal cancer was the most common primary tumour for liver metastases in 72/109 patients; for further details see Table 2. Additional therapies applied before and after RFA are listed in Table 3. Median follow-up was 56.8 months. In summery 178 MR guided RFA interventions were performed.

**Table 3:** Overview of the applied therapies before and after RFA

Patients (total *n* = 109)	Before RFA	After RFA
Surgery of the primary tumour	33	3
Surgery of the primary tumour + CTX	36	11
CTX	4	36
Surgery of the primary tumour + liver surgery	9	2
liver surgery	–	5
Surgery of the primary tumour + RCT	3	1
Surgery of the primary tumour + liver surgery + CTX + RCT	3	–
Surgery of the primary tumour + liver surgery + CTX	8	1
liver surgery + CTX	–	6
Surgery of the primary tumour + liver surgery + CTX + RT	1	–
Surgery of the primary tumour + CTX + RT	–	2
Surgery of the primary tumour + liver surgery + RCT	1	–
Surgery of the primary tumour + CTX + RCT	5	1
CTX + RCT	–	2
Surgery of the primary tumour + CTX + RT	5	–
CTX + RT	1	4
RT	–	2
Unclear	–	21
No further therapy	–	12

*CTX* chemotherapy, *RCT* radiochemotherapy, *RT* radiotherapy

### Overall survival

#### Whole study group of hepatic metastases of different tumour entities (*n* = 109 patients)

**Fig. 2:**
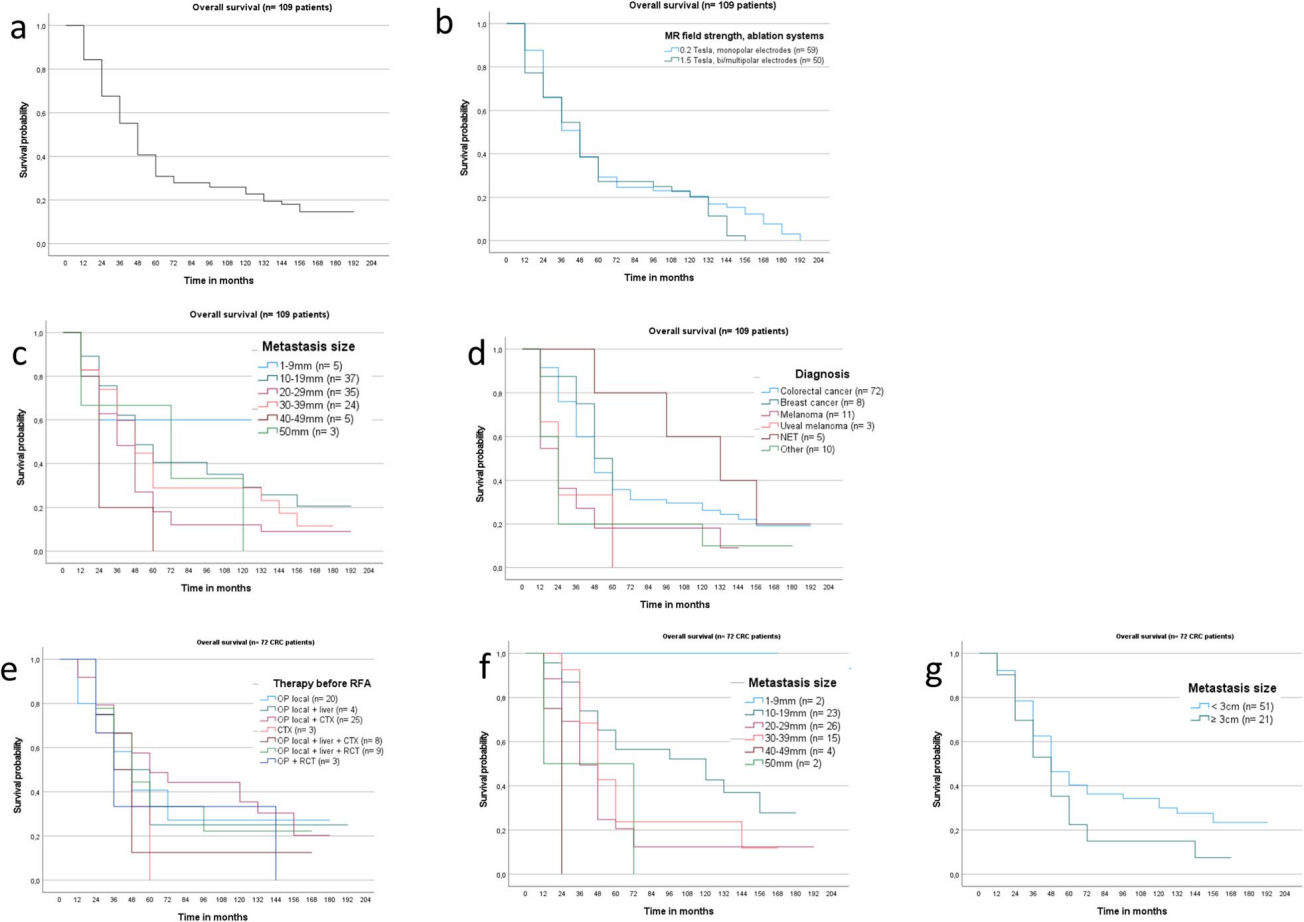
Kaplan–Meier curve for OS of the whole study cohort (**a** *n* = 109 patients, median 39.2 months, SD 3.7) and respectively for procedures performed with monopolar electrodes at 0.2 T and bi/multipolar electrodes at 1.5 T (**b** median 36.5 vs. 37.5 months; 95% CI respectively 28.3–44.6 and 25.7–49.3). RFA treated lesions up to 9 mm revealed the highest OS (**c** median not reached). OS according to the primary tumours, revealed significant differences with highest OS for patients with NET (**d** median 130.5 months, SD 46.4, 95% CI 39.4–221.5; *p* = 0.026) and lowest survival rates for uveal melanoma patients (median 15.0 months, SD 3.8, 95% CI 7.5–22.5). In 72 CRC patients, highest OS was documented for therapy combination of surgery and chemotherapy (**e** median 53.0 months, SD 16.1; 95% CI 15.5–90.5) and lesions up to 19 mm (**f**). No significant OS differences were detected between treated lesions smaller or larger than 30 mm (**g** *p* = 0.183)

Median survival rate of the whole study group was 39.2 months (SD 3.7, 95% CI 31.9–46.5). The 1- 3- 5- and 10-year overall survival rates of the whole patient cohort were 83.4%, 53.4%, 31.0% and 22.9% (SD 3.6, 4.8, 4.5 and 4.1; Fig. 2a). The MR field strength and the applied ablation system did not influence the overall survival of the whole study cohort (Fig. 2b).

According to RFA treated size of the liver metastasis, survival decreased from median 44.3 months (lesions diameter between 10 and 19 mm, 95% CI 57.6–99.2) to 22.8 months (lesions diameter 40–49 mm, 95% CI 8.2–42.0). However, the largest lesions displayed highest survival with 66.3 months (lesions of 50 mm; 95% CI 0.5–120.7; Fig. 2c) owing to a small sample size of three patients. Significantly higher OS rates were gathered for patients with NET (*p* = 0.03; Fig. 2d).

#### Patients with colorectal cancer (*n* = 72 patients)

Median survival rate was 39.5 months (SD 4.5; 95% CI 30.7–48.4). The 1- 3- 5- and 10-year overall survival rates of colorectal cancer patients were 90.2%, 57.1%, 36.1% and 26.5% (SD 3.5, 5.9, 5.8 and 5.4; Fig. 2d).

Highest median OS was detected in CRC patients, who received surgery of the primary tumour and chemotherapy before RFA (53.0 months, SD 19.1, 95% CI 15.5–90.5; Fig. 2e), lowest median survival for patients with previous surgery of the primary tumour and liver surgery (25.6 months, SD 14.7; 95% CI 0.0–54.5). According to size of the treated liver metastasis, highest OS data were detected for lesions between 1 and 19 mm with a survival of 28.5% after 145 months (median not reached; Fig. 2e). No significant differences in OS were detected in patients with lesions up to 29 mm in comparison to larger RFA treated liver metastases (*p* = 0.183; Fig. 2g).

### Progression free survival

#### Whole study cohort of hepatic metastases of different tumour entities (*n* = 109 patients)

**Fig. 3:**
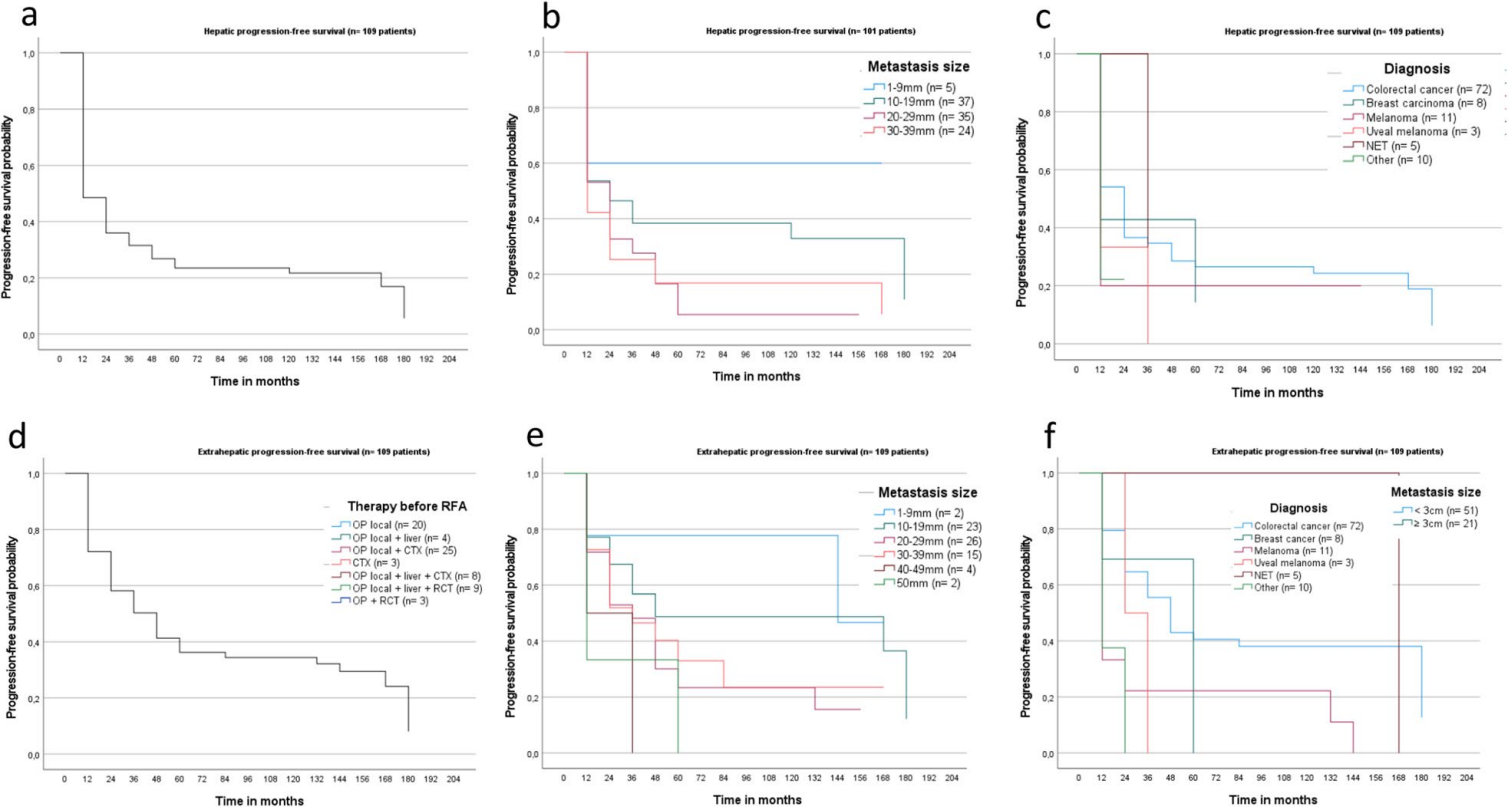
Kaplan–Meier curve depicts hepatic PFS of the whole study cohort in 73/109 patients (66.9%; **a**). Thereof, RFA treated lesions up to 29 mm, revealed longer hepatic PFS, compared to lesions over 30 mm (**b** however not statistically significant *p* = 0.092). Patients with NET and CRC revealed the highest hepatic PFS (**c** median 24.2 and 13.0 months, SD 3.9, 95% CI 5.2–20.9). Lowest hepatic PFS showed melanoma patients (**c** median 4.1 months, SD 2.1; 95% CI 0.1–8.2). Extrahepatic PFS of the whole study cohort occurred after median 37.0 months (**d** SD 9.3; 95% CI 18.7–55.2). Thereof highest extrahepatic PFS was documented for RFA-treated lesions up to 9 mm (**e** median 135.2 months, SD 96.2; 95% CI 0–323.9) and for patients with NET (**f** median 156.5 months)

Metastatic liver lesions, were detected in 73/109 patients (66.9%) after median 10.2 months (SD 1.6; 95% CI 6.9–13.5; Fig. 3a). According to the OS data, a larger size of the initially RFA-treated liver lesion, resulted in shorter hepatic PFS (Fig. 3b). The highest hepatic PFS rates showed patients, suffering from NET (median 24.2 months) and colorectal cancer (median 13.0 months, SD 3.9; 95% CI 5.2–20.9; Fig. 3c).

Extrahepatic metastases were diagnosed after median 37.0 months (SD 9.3, 95% CI 18.7–55.2; Fig. 3d). Highest extrahepatic PFS was obtained for RFA-treated lesions up to 9 mm (median 135.2 months; SD 96.2; 95% CI 0–323.9), with no significant differences between RFA-treated lesions up to 29 mm and larger ones (*p* = 0.177, Fig. 3e). According to the primary diagnoses, highest extrahepatic PFS were found in patients suffering from NET, breast cancer and CRC (respectively median 156.5, 48.4 and 44.1 months, Fig. 3f).

#### Patients with colorectal cancer (*n* = 72 patients)

**Fig. 4:**
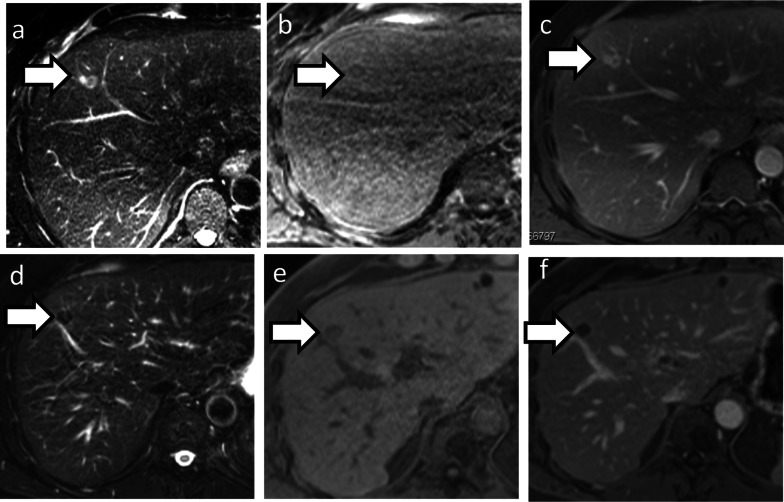
Hepatic metastasis in segment IV in a 55-year old patient suffering from rectal cancer with further pulmonary metastasis (**a** T2w, **b** T1w, **c** T1w post contrast media application). After radiofrequency therapy of the liver, complete response was achieved without any disease recurrence 15 years later (**d** T2w, **e** T1w, **f** T1w post contrast media application)

**Fig. 5:**
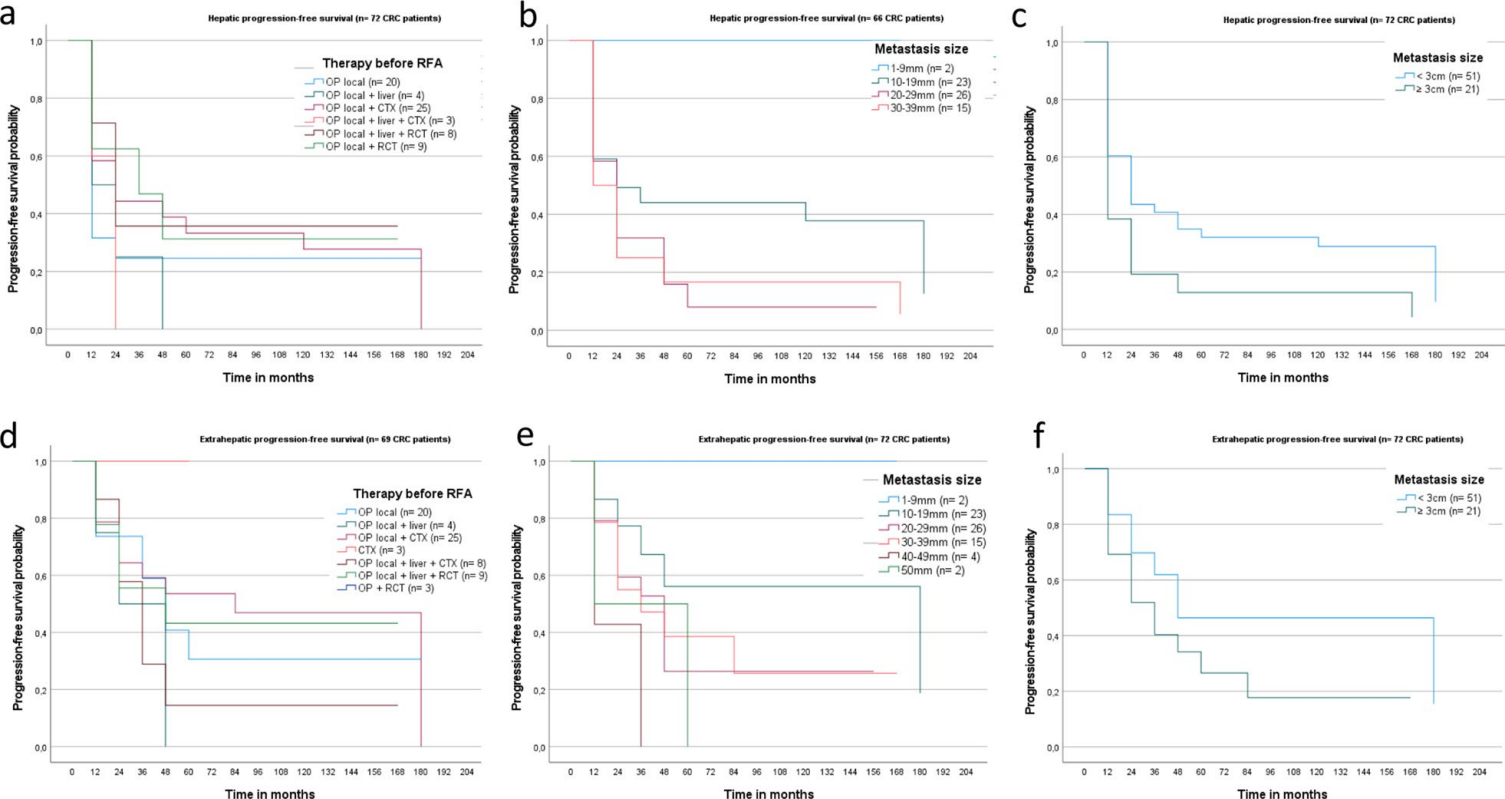
Kaplan–Meier curve shows superior hepatic PFS in 72 CRC patients for surgical treatment in combination with CTX, compared to surgery alone (**a** median 14.4 and 6.6 months, SD 5.1, 1.4; 95% CI 4.4–24.5 and 3.8–9.4). Hepatic PFS was significantly higher for RFA treated liver lesions up to 29 mm compared to larger lesions (**b**, **c** median 19.0 months vs. 7.8 months; SD 2.7 and 3.3, 95% CI 13.7–24.4 and 1.2–14.4; *p* = 0.035). Extrahepatic PFS in CRC patients revealed no significant differences according to either the performed therapy before RFA (**d** *p* = 0.580) or the lesion size of the RFA-treated metastases (**e**, **f** *p* = 0.067)

Median hepatic PFS of CRC patients was 13.0 months (SD 3.9; 95% CI 5.2–20.9; Fig. 4). Patients treated with solitary surgery of the primary tumour, had shorter hepatic PFS than patients treated with additional chemotherapy (*n* = 20 vs. 25; median 6.6 months vs. 14.4 months; SD and 95% CI respectively 1.4, 5.1 and 3.8–9.4, 4.4–24.5; *p* = 0.199; Fig. 5a). Longer hepatic PFS was gained for RFA-treated liver lesions up to 29 mm in comparison to larger ones (median 45.1 vs. 25.1 months, SD and 95% CI respectively 18.8, 10.2 and 8.2–82.1 and 5.0–45.3), however not statistically significant (*p* = 0.062; Fig. 5b, c).

Median extrahepatic PFS was 44.1 months (SD 7.0; 95% CI 30.4–57.9). No significant differences according to the performed therapies before RFA (*p* = 0.58; Fig. 5d) or the lesion size of the initially RFA-treated metastases were detected (*p* = 0.067, Fig. 5e, f).

### Patient reported outcome (PRO)

28/109 patients were either directly or via relatives contacted for assessment of the patient reported outcome (PRO). RFA was memorable less incriminatory than other applied therapies for 15 patients, whereas four patients remembered RFA to be more stressful compared to chemotherapy (*n* = 3) or surgery (*n* = 1). In nine patients RFA procedure could not be retrospectively classified in relation the other therapies.

Self-sustaining way of life, was either reported from the patients or documented in patients’ records in 60 patients after RFA procedure, whereas nine patients were in need of care. Another 40 patients’ data were not evaluable due to the long survey period of data analysis.

## Discussion

This single-centre study reports long-time survival rates of at least 10 years after MR-based RFA of liver metastases from different tumour entities. With a median OS of 39 months from the whole study cohort of 109 patients, indifferent of the primary tumour type, and 1- 3- and 5-year OS of 83%, 53% and 31%, our results is comparable to literature data (86%, 44% and 31% [15]). In contrast to Liu, we obtained significantly different survival rates according to the primary tumour, with highest OS rates for patients with neuroendocrine tumours (NET; *p* 0.02; median 130 months, 5-year OS 60%). Although the indication of liver treatment with RFA in NET patients is still a matter of debate, our long-term 10-year OS of 40% in five patients is in line with surgically and ultrasound-guided performed RFA [16]. Even though our 10-year single-centre OS rate of the whole study group of 22% is not comparable to surgical OS data, due to the variety of included primary tumours, it serves as a first documentation for future survival analysis.

We focused our analysis on percutaneously, RFA-treated liver metastasis of 72 colorectal cancer (CRC) patients, due to the high prevalence of 50–60% for metastases in this tumour entity, thereof 80–90% non-resectable liver metastases [17]. Our single centre CRC patient cohort revealed with 39 months a higher median OS, compared to a recent literature review with 30 months [18]. One reason might lie in the high rate of complementary CTX for treatment of micrometastases [19], documented in our cohort before and after RFA (34% and 47%), as extrahepatic tumour spread together with new liver lesions are the main cause for death in RFA treated CRC patients [20]. Furthermore, issues like tumour aggressiveness, patient selection as well as the physicians experience are reflected in patient’s outcome [2], which are difficult to compare with other published studies.

High rates of local recurrences after RFA are reported for incomplete treated metastases, located near large vessels [18], or larger than 3 cm in size [21]. Although analysis of local disease recurrence was not the focus of this study, we obtained significantly shorter hepatic PFS in CRC patients with metastases over 3 cm, compared to smaller lesions. Compared to surgery, shorter hepatic PFS is reported for patients treated with RFA in the liver, although OS is similar [22, 23]. However, for a detailed comparable survival analysis of treated liver metastases (surgical vs. minimal invasive via RFA under MR, CT, US or laparoscopic guidance) a prospective multi-centre register should be established. This register should include a prospective patient survey regarding the invasiveness of the performed method and a patient subjective comparative analysis to other complimentary performed therapies like chemotherapy or radiation therapy. Based on these data, a detailed evaluation of the survival data regarding the applied therapeutic procedures, including an analysis of quality-adjusted life years (QUALY) is possible [24]. Such a prospective register provides even the possibility for a cost-effectiveness analysis, as already performed for melanoma patients in diagnostic imaging methods like PET/CT [25].

Limitations of this study encompass its single-centre design with an overlap of analysed survival data from other studies [12, 26] as well as the RFA procedures performed at 0.2 T. However, our overall survival data reveal no relevant difference between patients with RFA procedures at 0.2 T and 1.5 T and other studies also described the feasibility of RFA at 0.2 T [5]. Furthermore, the retrospective and long-term study character impairs the analysis of numerous patient-reported outcomes. Those should be addressed via a prospective questionnaire directly after each performed RFA in the future. Additionally, the comparability of long-term survival data of the whole study cohort to other studies is limited, as a multitude of primary tumours lead to hepatic metastases. However, our focus on CRC patients as the largest subgroup in our study cohort, overcomes this impairment.

In conclusion this is the first single centre, long-term outcome analysis of percutaneously, MR-guided performed RFA of liver metastases of different tumour entities. Future work should focus on a simultaneous data collection of patients’ experiences to the intervention time point for representative analysis.

## Data Availability

The datasets used and/or analysed during the current study are available from the corresponding author on reasonable request.

## Abbreviations

CI: Confidence interval
CRC: Colorectal carcinoma
CT: Computed tomography
CTX: Chemotherapy
DWI: Diffusion weighted imaging
FISP: Fast imaging with steady precession
FLASH: Fast low angle shot gradient echo sequence
HASTE: Half acquisition single shot turbo spin echo sequence
MRI: Magnetic resonance imaging
MWA: Microwave ablation
NET: Neuroendocrine tumour
OS: Overall survival
PFS: Progression free survival
PRO: Patient reported outcome
QUALY: Quality-adjusted life years
RCT: Radio-chemotherapy
RFA: Radiofrequency ablation
SSFP: Steady-state free precession sequence
TSE: Turbo spin echo sequence
US: Ultrasound
VIBE: Volumetric interpolated breath-hold examination
